# Approximate Hamiltonians from a Linear Vibronic Coupling Model for Solution-Phase Spin Dynamics

**DOI:** 10.1021/acs.jctc.4c01437

**Published:** 2025-01-17

**Authors:** Toby R. C. Thompson, Jakob K. Staab, Nicholas F. Chilton

**Affiliations:** † Department of Chemistry, The University of Manchester, Manchester M13 9PL, U.K.; ‡ Research School of Chemistry, Australian National University, Canberra, Australian Capital Territory 2601, Australia; § Department of Chemistry “Ugo Schiff”, INSTM Research Unit, Universitá degli Studi di Firenze, 50019 Sesto Fiorentino, Italy

## Abstract

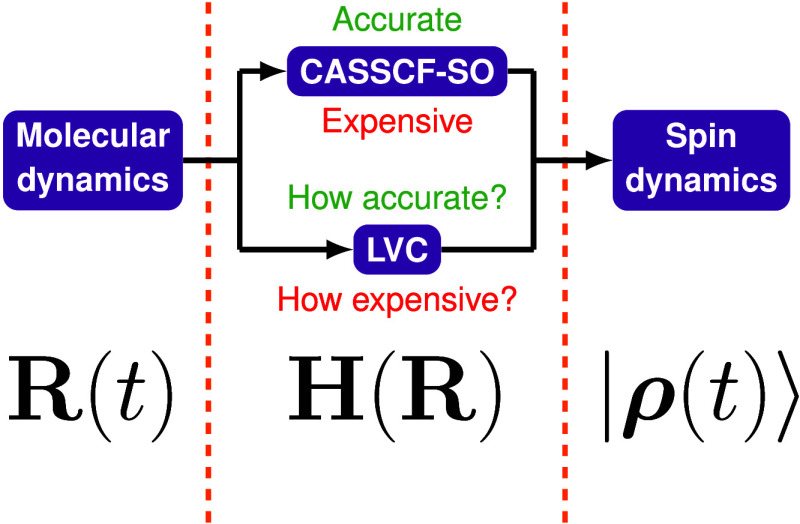

The linear vibronic coupling (LVC) model is an approach for approximating how a molecular Hamiltonian changes in response to small changes in molecular geometry. The LVC framework thus has the ability to approximate molecular Hamiltonians at low computational expense but with quality approaching multiconfigurational *ab initio* calculations, when the change in geometry compared to the reference calculation used to parametrize it is small. Here, we show how the LVC approach can be used to project approximate spin Hamiltonians of a solvated lanthanide complex along a room-temperature molecular dynamics trajectory. As expected, the LVC approximation is less accurate as the geometry diverges from that at which the model was parametrized. We examine the accuracy of the predicted Hamiltonians by performing time-dependent quantum simulations of the spin dynamics of the molecule, with reference to the dynamics obtained using spin Hamiltonians projected from *ab initio* calculations at each step. We find that quantitatively accurate behavior is obtained when LVC parametrizations are performed at least every 10 fs during the trajectory.

## 1 Introduction

Knowledge of a chemical system’s Hamiltonian operator is essential for describing both static properties and its evolution through time.^1^ The complete electronic Hamiltonian for a general molecule, where significant orbital degeneracies and spin–orbit coupling (SOC) may be present, can often be well approximated using multiconfigurational methods such as complete active space self-consistent field spin–orbit (CASSCF-SO) calculations, but these approaches require significant computational resources. While performing such calculations on static structures is commonplace, problems that require evaluation of the molecular Hamiltonian at this level of theory for many nuclear configurations remain challenging. These include excited-state dynamics, in which the energies of and couplings between multiple states must be considered.^2−4^ Ground-state spin dynamics in metal complexes can also be simulated using knowledge of the Hamiltonian at many molecular dynamics (MD) timesteps.^5,6^ In principle, the prediction of a great variety of properties can be improved if the Hamiltonian and its eigenstates are known at many molecular geometries, by computing a weighted average.^7^

**Figure 1:**
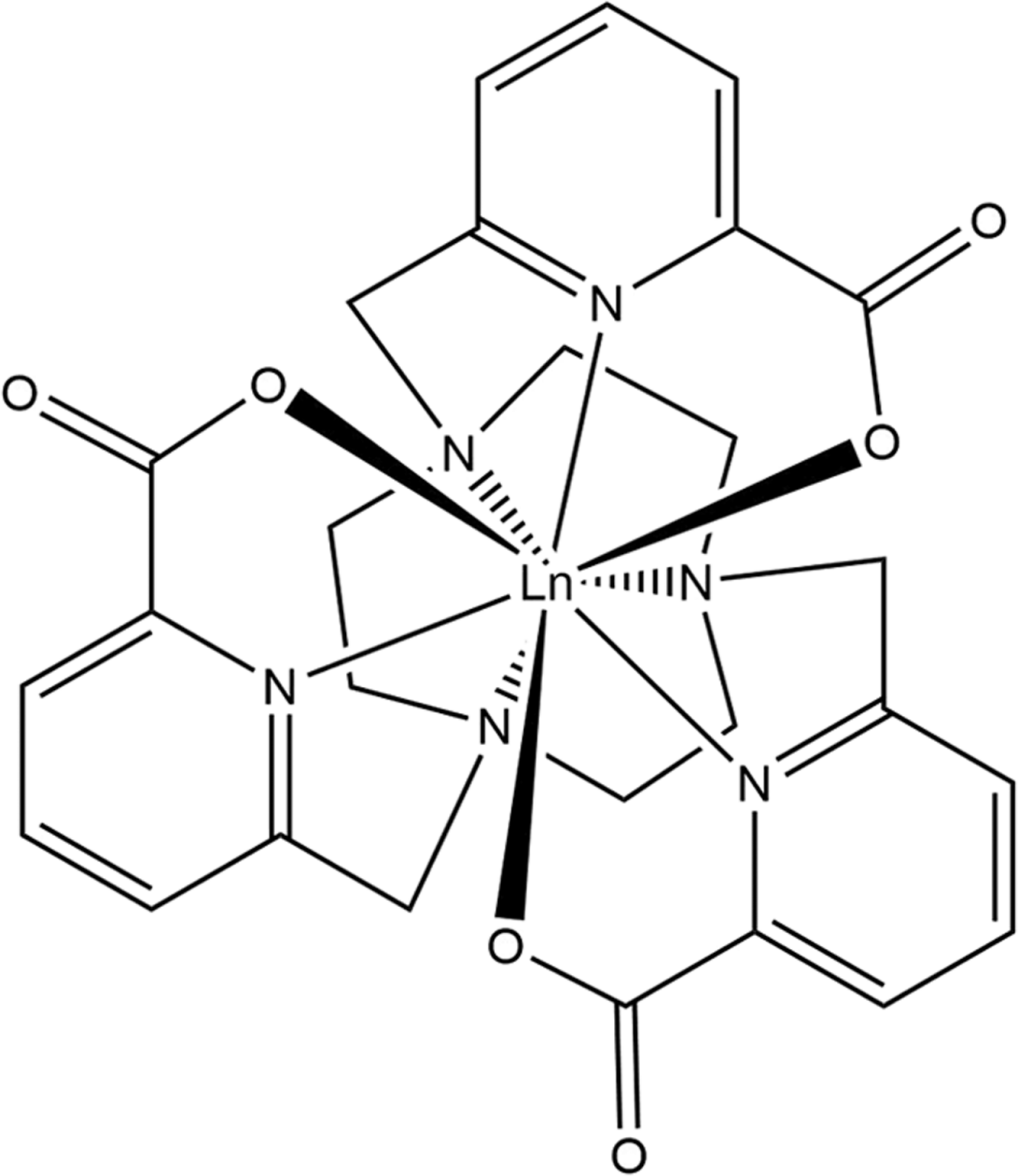
Molecular structure of [LnL^1^].

In a previous study,^7^*ab initio* MD (AIMD) simulations of a lanthanide complex ([GdL^1^], Figure 1) in solution were performed. [LnL^1^] is a frequently studied^7−12^ member of the PARASHIFT family of magnetic resonance imaging agents, complexes with the ability to reveal information on temperature and pH *in vivo*.^13,14^ [DyL^1^] in particular has attracted attention due to its unusual pseudocontact shift behavior. The equatorial crystal field potential due to the pyridyl donor atoms approximately cancels the axial potential arising due to the amine donor atoms, leading to a crystal field that is dominated by, and extremely sensitive to the positions of, the carboxylate oxygen atoms. In the molecular structure, these oxygen atoms happen to sit extremely close to the magic angle (where the leading crystal field parameter *B*_2_^0^ changes sign in simple point-charge crystal field theory, corresponding to a change from an axial to an equatorial type crystal field potential), such that even very small movements can change the sign of the magnetic anisotropy, and hence also the signs of the pseudocontact shifts of nuclei in the ligands. This leads to wildly different pseudocontact shifts for seemingly indistinguishable structures found by different optimization methods, and generally poor agreement with experimental NMR shifts when using static structures.^11^ Time-averaged ^1^H NMR shifts, determined on the basis of Gd^3+^ AIMD trajectories with the metal replaced by Dy^3+^, were in much closer agreement with experiment but had to be obtained from CASSCF-SO calculations performed at regular intervals throughout the trajectories. Even the solvent-dependence of the paramagnetic shifts observed experimentally was captured using this AIMD + CASSCF-SO approach.

The function of PARASHIFT agents relies on the acceleration of nuclear spin relaxation for signal enhancement, which occurs due to the interaction with the electron spin dynamics–understanding these dynamics is thus key to the design and use of such imaging agents. Our AIMD simulations offer an opportunity to simulate the electron spin dynamics in a PARASHIFT agent from first principles. However, describing spin dynamics in solution-state metal complexes with significant spin–orbit coupling is a challenging and long-standing problem. It is difficult primarily because anisotropic lanthanide ions have a strong ligand field contribution to their molecular Hamiltonian that dominates the spin quantization, and thus the time-varying ligand field due to the internal motions of the complex is the main driver of the electron spin dynamics. To make matters worse, the high-field and motional-narrowing assumptions are invalid for electron spin dynamics in such systems.^15−17^ Hence, explicit time-domain dynamics must be simulated using a time-ordered series of Hamiltonians, but, as described above, generating such a set of Hamiltonians is a daunting computational task. Previous approaches have either relied on simplified phenomenological models^18,19^ or incurred errors due to the computational expense of performing electronic structure calculations at frequent enough intervals throughout an MD trajectory.^5,6,20,21^ Thus, an approach that is computationally cheaper than a full stack of *ab initio* CASSCF-SO calculations but can still capture the fs-scale detail inherent to an MD trajectory is desirable. Recent literature has focused on machine learning as a computationally approachable means of generating high-accuracy molecular dynamics trajectories,^22−24^ obtaining spin Hamiltonian parameters^25−27^ and even performing spin dynamics simulations themselves,^28^ on long time scales. However, the field is not yet mature to the point of accessible, highly transferable implementations being available for spin dynamics applications. We thus turn our attention to the possibility of a computationally cheap MD-driven spin dynamics methodology rooted in established electronic structure methods, specifically the linear vibronic coupling (LVC) model.

The LVC approach uses an *ab initio* electronic structure calculation at a particular geometry to parametrize a model that can be used to approximate the molecular Hamiltonian at a nearby geometry.^2,29^ It takes a diabatic view, with couplings between states taken into account, and is compatible with SOC. The LVC methodology has been successfully applied in the domain of excited-state dynamics, allowing qualitative agreement with population dynamics from models 3 orders of magnitude more expensive,^2^ as well as with experimental absorption and electronic circular dichroism spectra by explicit wavepacket propagation, for molecules with significant vibrationally mediated interstate couplings.^30^ It is also in competition with machine learning for excited-state dynamics simulations.^31,32^ It has also been successfully applied to electron spin relaxation in single-molecule magnets, where it is employed to obtain analytical functional derivatives of the molecular Hamiltonian, or of spin Hamiltonian parameters.^29,33^ A single parametrization can in principle be used to generate approximate Hamiltonians at many points along an MD trajectory, allowing spin dynamics simulations to be carried out at potentially lower cost than with an electronic structure calculation at each time step. However, the error in an LVC-generated Hamiltonian is expected to increase as the geometry it is evaluated at diverges from the geometry the model was parametrized at–an investigation of this error and its effects on the simulated spin dynamics is required before practical applications are possible.

## 2 Methods

### 2.1 Linear Vibronic Coupling

The full molecular Hamiltonian, as computed during a CASSCF-SO calculation, can be divided into two components:
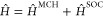
1where *Ĥ*^MCH^ is the molecular Coulomb Hamiltonian (MCH), containing only spin-free operators, and *Ĥ*^SOC^ is the SOC Hamiltonian. In the diabatic picture, the (diagonalized) matrix form of *Ĥ*^MCH^ can be expanded to first order in the molecule’s nuclear coordinates **R**. This permits a linear approximation of the MCH from the reference geometry **R** = **0** to another similar geometry **R** = Δ**R**
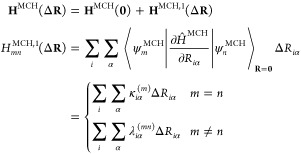
2where ψ_*m*_^MCH^ are the eigenstates of *Ĥ*^MCH^ at **R** = **0**. The indices *m* and *n* run over all spin-free states for a given spin multiplicity considered in *Ĥ*^MCH^, *i* runs over all nuclei and α ∈ {*x*, *y*, *z*}. **κ**^(*m*)^ and **λ**^(*mn*)^ are the gradients and nonadiabatic coupling (NAC) coefficients, respectively, which can be found following a multiconfigurational electronic structure calculation.^34,35^

The molecular Coulomb Hamiltonian at distorted geometry **H**^MCH^(Δ**R**) can be diagonalized, yielding energies and expressions for the new eigenstates in terms of the eigenstates of **H**^MCH^(**0**) (i.e., the mixing of states caused by the change in geometry). The same unitary transformation that diagonalizes **H**^MCH^(Δ**R**) can be applied to the matrix representation of any operator evaluated in the eigenbasis of **H**^MCH^(**0**), generating the equivalent operator in the eigenbasis of **H**^MCH^(Δ**R**).

The atomic mean-field integral (AMFI) method^36^ approximates the SOC Hamiltonian as
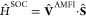
3where **V̂**^AMFI^ is a spin-free operator evaluated in its matrix form from integrals over atomic basis functions, and **Ŝ** is the electron spin operator. **V**^AMFI^(**0**) can be transformed into the eigenbasis of **H**^MCH^(Δ**R**) as previously described, allowing **H**^SOC^(Δ**R**) to be constructed via the Wigner-Eckart theorem as it would be during a standard CASSCF-SO calculation.^29^ We are thus assuming that *Ĥ*^SOC^ is not itself inherently geometry dependent–this approximation has recently been reported to break down for a Dy^3+^ complex^37^ but appears to be reasonable for [LnL^1^] (see Supporting Information (SI), Figure S2). Such geometry dependence could in principle be taken into account via derivatives of the AMFI integrals. All terms present in **H**(Δ**R**), the full molecular Hamiltonian at distorted geometry, are now known.

### 2.2 Electronic Structure

Prior to AIMD simulations, molecular mechanics-driven MD was performed to equilibrate a simulation box with sides of 20 Å containing only CD_3_OD molecules. A deuterated, geometry-optimized [GdL^1^] complex was then inserted into the center. This was followed by optimization of both atomic positions and the periodic box’s volume. AIMD simulations were then carried out using VASP 6.2.0.^38^ The NVT ensemble was employed with a time step of 1 fs and a temperature of 300 K. Forces were found using density functional theory, specifically the PBE^39^ exchange-correlation functional with Grimme’s D3 dispersion correction^40^ and an f-in-core potential for Gd^3+^. For full details of the MD methodology see the original study.^7^

Dysprosium-based PARASHIFT agents are of interest due to their large pseudocontact shifts–these maximize separation of the complex’s proton resonances from those of water and fat in the body. The acceleration of nuclear relaxation in Dy^3+^ complexes is also often more significant than that of other viable lanthanide ions, improving signal intensity.^13,17^ Accordingly, state-averaged CASSCF-SO calculations and LVC parametrizations were performed on snapshots from these AIMD simulations using OpenMolcas 23.02,^41^ with Gd^3+^ replaced by Dy^3+^. The active space was nine electrons in the seven 4f orbitals, and the 18 lowest roots of the ground 5/2 spin state, corresponding to the ^6^H and ^6^F terms, were considered with equal weighting. The ANO-RCC-VTZP basis set was employed for dysprosium, the ANO-RCC-VDZP basis set for the ligand atoms directly adjacent to the metal and the ANO-RCC-VDZ basis set for the other ligand atoms.^42^ Two-electron integrals were decomposed using the atomic compact Cholesky method.^43^ SOC was taken into account via the AMFI approximation.^36^ Scalar relativistic effects were treated with the second order Douglas-Kroll Hamiltonian.^42,44^

The explicit solvent molecules present in the MD simulation were included in each CASSCF-SO calculation as point charges. This allowed the motion of the solvent to be considered by the LVC model. These charges were found from a DFT geometry optimization (PBE^39^ functional and cc-pVDZ basis set^45^) and CHELPG decomposition^46^ of a single methanol molecule in vacuum using Gaussian 16.^47^ As the solvent molecules consisted only of point charges, those split across the AIMD simulation’s periodic boundary were not made whole. When applying the LVC model, solvent atoms that had crossed the boundary since the parametrization had the potential to lead to significant errors due to this large change in geometry. Any boundary-crossing atoms were translated to their position in an adjacent periodic image, such that their displacement from the parametrization geometry was minimized. The Kirkwood continuum model^48^ was also employed during CASSCF-SO calculations to factor in long-range solvent effects (truncated at first order, using the experimentally measured dielectric constant for methanol of 33.3^49^). This places the system within a spherical cavity surrounded by a dielectric; the dielectric is polarized by the system, generating multipoles (in this case no terms beyond dipole are included) that effect the system in turn. The radius of this sphere was chosen to be large enough to include the entire simulation box.

Within the spin Hamiltonian formalism, the electron spin states of a lanthanide complex in the absence of an external magnetic field can be described with SOC and ligand field terms,^17^ the latter of which is expressed here as an effective crystal field in the Stevens formalism^50,51^
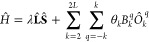
4where **L̂** is the electron orbital angular momentum operator, λ is the SOC parameter, *Ô*_*k*_^*q*^ are linear combinations of orbital angular momentum operators, θ_*k*_ are constants specific to an electron configuration and *B*_*k*_^*q*^ are the crystal field parameters. λ and *B*_*k*_^*q*^ can be projected so that the matrix elements of the spin Hamiltonian replicate those of the full molecular Hamiltonian in the same basis. The LVC method thus offers on opportunity to generate spin Hamiltonian parameters at many geometries (MD timesteps) with relatively few *ab initio* calculations, potentially reducing the computational cost of acquiring a series of Hamiltonians for electron spin dynamics simulations. It is also possible to use LVC to perform these simulations without projection, using the molecular Hamiltonian in the relevant angular momentum basis directly, however the spin Hamiltonian parameters lend themselves to a more intuitive analysis (*vide infra*) and their use is standard in this field.^5,6^ Spin Hamiltonian parameters for use in electron spin dynamics simulations were projected for the ^6^H term in the |*LSM*_*L*_*M*_*S*_⟩ basis.

### 2.3 Spin Dynamics

Spin dynamics simulations utilized the vectorised density matrix |**ρ**(*t*)⟩.^52^ This propagates through time according to the vectorised Liouville-von Neumann equation
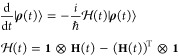
5where ⊗ denotes a Kronecker product and the identity matrix **1** has the same dimensions as **H**. In this work we have a piecewise-constant series of Hamiltonians found at MD timesteps separated by δ*t* = 1 fs. This allows the vectorised density matrix to be propagated in discrete steps
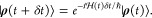
6This propagation made use of the matrix exponential-times-vector algorithm described in a recent monograph.^52^ This calculates |**ρ**(*t* + δ*t*)⟩ from eq 6 by expanding the matrix exponential as a Taylor series
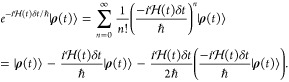
7Higher order terms, each found by applying  to the previous term, are successively added to |**ρ**(*t*)⟩ until the next term would not change any element by more than an arbitrarily small cutoff. Monotonic convergence of the Taylor series is guaranteed by dividing each time step into a series of equal-length substeps, each featuring evolution under the same Hamiltonian. The number of substeps is equal to the infinity-norm of the exponent, rounded to the next largest whole number; this algorithm reliably maintained Tr(**ρ**(*t*)) = 1 during spin dynamics trajectories (Figure S4).

While the Hamiltonian operators were initially evaluated in the |*LSM*_*L*_*M*_*S*_⟩ basis, the dynamics themselves were performed in the eigenbasis of the initial spin Hamiltonian at *t* = 0. This allowed simulations to start from a thermal density matrix **ρ**(0), with off-diagonal elements set to zero and populations taken from a Boltzmann distribution over the initial Hamiltonian’s eigenvalues. Despite the use of an initially thermalized density matrix, the dynamics calculated here do not give a realistic representation of the ensemble of systems observed during a magnetic resonance experiment, as they are entirely unitary and do not feature relaxation or inhomogeneous broadening. This makes the use of a vectorised density matrix strictly unnecessary when compared to a simpler wave function-based approach. The density matrix formalism has been applied nevertheless, as it leaves this methodology open to potential dissipative dynamics simulations in the future.

## 3 Results and Discussion

**Figure 2:**
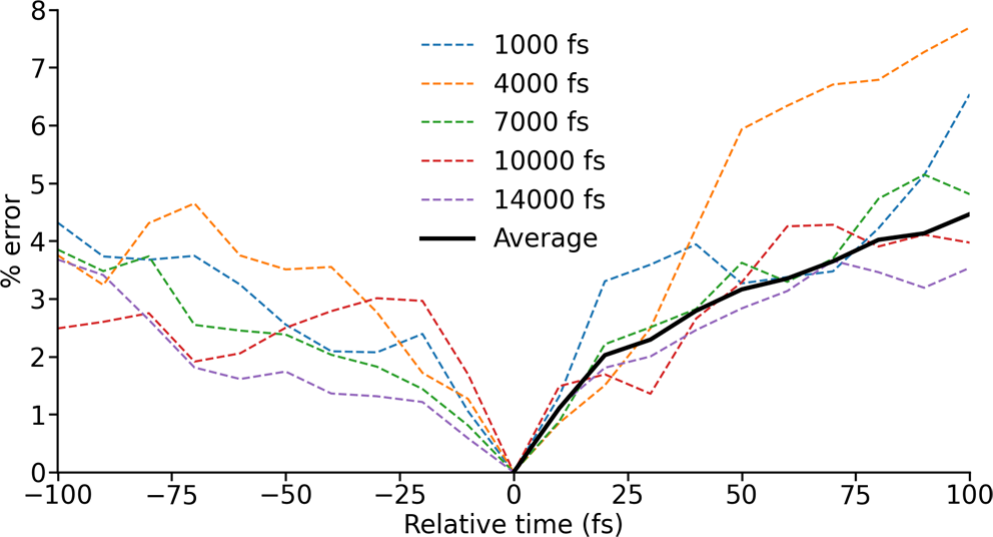
Error in an LVC-generated spin Hamiltonian as a function of the separation between the parametrization (relative time 0 fs) and the time at which the spin Hamiltonian is evaluated. This is plotted with the LVC parametrization carried out at a range of different times throughout an MD trajectory, and as an average taking into both account both positive and negative relative time.

Equation 2 is simply a Taylor series truncated at first order, and as such the LVC model incurs a truncation error that is expected to increase as the geometries **R** = **0** and **R** = Δ**R** diverge. The error in an LVC-generated spin Hamiltonian can be assessed by comparison to that from an explicit CASSCF-SO calculation at the same geometry
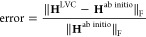
8where ∥∥_F_ denotes the Frobenius norm. We picked a set of five starting points along the trajectory at which we parametrized an LVC model, and then we evaluated the error as a function of the distance between the timesteps used for parametrization and evaluation of **H**^LVC^ (Figure 2). As expected, the error increases for larger time differences between parametrization and evaluation, reflecting an increasing divergence in molecular geometry. The behavior of this error is similar throughout an MD simulation, and similar regardless of whether the evaluation is before or after the parametrization. These results justify the use of a series of LVC parametrizations performed at regular intervals throughout an MD simulation for the approximation of molecular Hamiltonians. The question hence becomes what is the largest acceptable interval between subsequent LVC parametrizations (i.e., what is the largest acceptable error) such that computational expense can be minimized?

**Figure 3:**
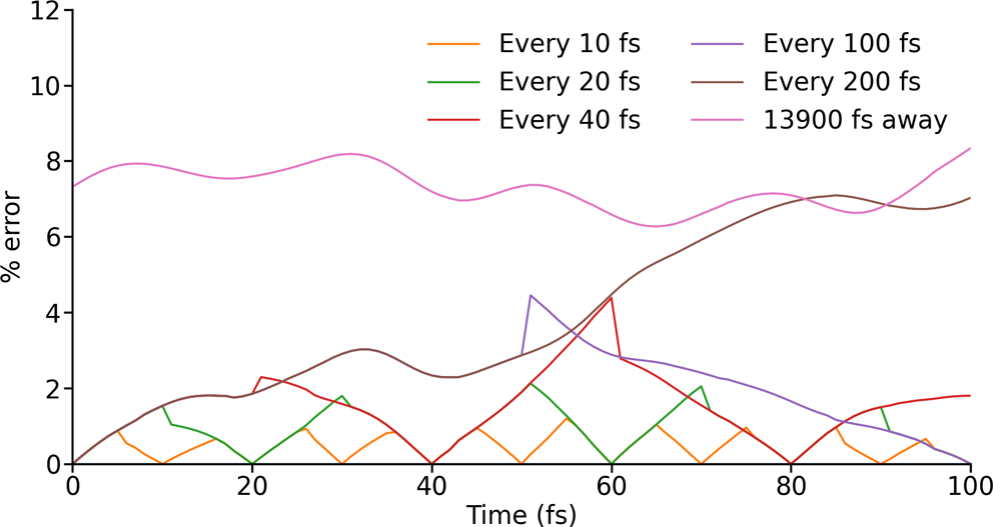
Error in an LVC-generated spin Hamiltonian over 100 fs with LVC parametrizations carried out at different intervals. 0 fs is the beginning of the error trajectory and the point at which the LVC model is first parametrized. In one case a single LVC parametrization is carried out at 13900 fs.

First, we must quantify the error incurred as a function of time using LVC models in a time-ordered geometry sequence. To do so, we generated spin Hamiltonians at every time step from 100 to 200 fs in the MD trajectory, using both explicit CASSCF-SO calculations and the LVC model with parametrizations carried out at different intervals, and quantified the error (Figure 3); we also made an LVC parametrization using the geometry at 14000 fs in order to estimate the largest possible error. As expected, the maximum error in **H**^LVC^ increases as the LVC parametrizations become further apart. Using only a single parametrization of the LVC model at the beginning of this window, henceforth referred to as 0 fs, the error reaches that for a very distant LVC model by 100 fs; at this limit, the error fluctuates between 6 and 9%.

The projection of spin Hamiltonian parameters allows Hamiltonians containing only one of the terms in eq 4 to be constructed, and their individual errors computed with eq 8 (see SI, Figures S1–S3). The SOC term leads to no significant error, even at large geometric deviation, as it varies so little over time that even the constant value of λ obtained from a single LVC parametrization remains accurate (this may not be the case if *Ĥ*^SOC^ in eq 1 changes significantly with geometry^37^). The crystal field term contributes less to the magnitude of **H** but varies much more. It becomes extremely inaccurate and is almost wholly responsible for the error seen in the total spin Hamiltonian. This is equivalent to the energy gaps between separate spin–orbit coupled multiplets remaining constant and being well reproduced, while those between the individual spin states within each multiplet vary and become increasingly inaccurate. It is exactly these variations that drive the electron spin dynamics of solution-state lanthanide complexes.^5,16,19^

The error can be reduced by parametrizing a new LVC model at intermediate points along the trajectory, and, following from the results above (Figure 2), we can use the new LVC model to move forward *and* backward. Hence, a reparameterisation at 100 fs can be used to predict Hamiltonians between 50 and 99 fs equally well as those for 101–150 fs; note, however, that in this particular case it appears the prediction based on the parametrization at 0 fs is better between 50 and 55 fs than that based on the parametrization at 100 fs. Thus, performing LVC parametrizations at increasingly dense bisections of the time-domain leads to an approximate halving in the maximum error as a function of time (Figure 3), although this becomes unreliable when the time between parametrizations is large.

We note that the use of a series of different LVC models leads to discontinuities in the error traces (Figure 3), which implies there must be discontinuities in the LVC-approximated Hamiltonians and spin Hamiltonian parameters (Figures S2 and S3), when one LVC parametrization is replaced by another. This is concerning, as it could lead to incorrect behavior during spin dynamics simulations. However, we show that Tr(**ρ**(*t*)) = 1 is maintained during simulations that feature these discontinuities (Figure S4), showing that such discontinuities do not prevent accurate spin dynamics simulations using the LVC model.

**Figure 4:**
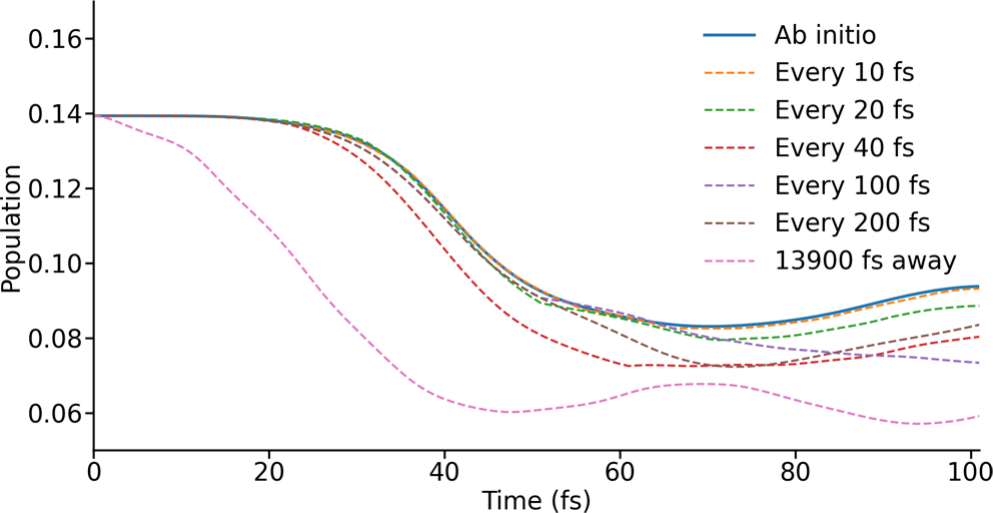
Population trajectories for one of the ground eigenstates of the initial spin Hamiltonian, found using spin Hamiltonians from *ab initio* calculations and the LVC model parametrized at different intervals. In one case a single LVC parametrization, carried out at 13900 fs, is used.

With knowledge of the error-scales due to the LVC model in hand, we turn now to explicit quantum simulations of the time-dependence of the electron spin using these series of Hamiltonians. As but one metric of these dynamics, we examine the time-dependent population of one of the states belonging to the ground Kramer’s doublet (Figure 4). The trajectory with the LVC model parametrized every 10 fs (**H**_10_^LVC^, corresponding to an error not exceeding 1%, Figure 3) behaves almost identically to that using only *ab initio*-calculated spin Hamiltonians, confirming that the LVC model can indeed be used to simulate spin dynamics with quantitative accuracy on this time scale. The approximate trajectory will eventually diverge, but as the dynamics of interest occur on the 100 fs time scale this is unlikely to have a significant effect. While moving to LVC parametrizations taken every 20 fs (**H**_20_^LVC^) might be acceptable in some cases, the population appears to diverge increasingly over 100 fs–hence, it may fail to give quantitatively accurate results for key properties such as spectral density functions.

**Figure 5:**
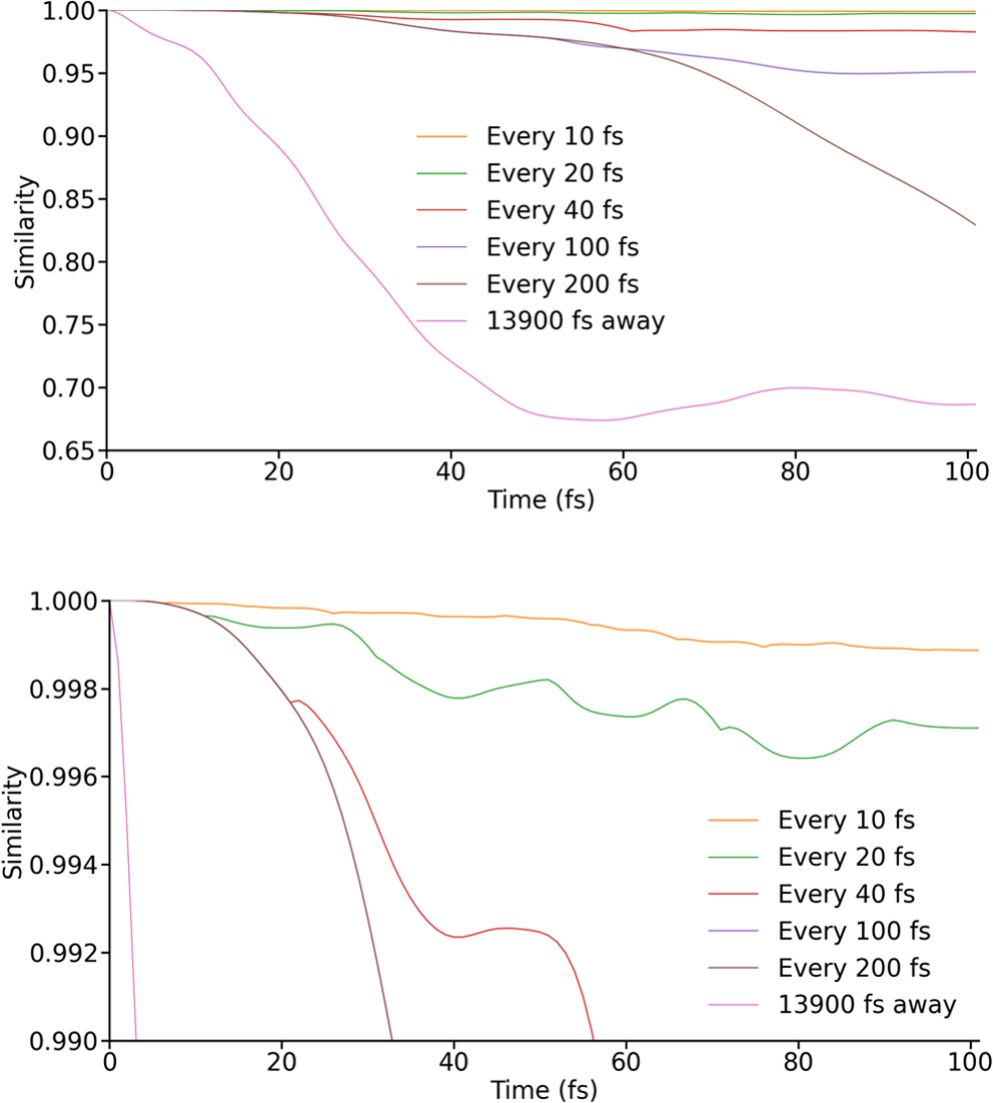
Similarity between the exactly propagated density matrix and those propagated using spin Hamiltonians from LVC models parametrized at different intervals. In one case a single LVC parametrization, carried out at 13,900 fs, is used. The lower image has been scaled to demonstrate the divergence of even the most accurate LVC-propagated trajectories.

**H**_40_^LVC^ clearly diverges from the exact result quite early in the simulation, around 30 fs, however, interestingly, models parametrized at longer intervals (e.g., **H**_100_^LVC^ and **H**_200_^LVC^) are able to maintain accuracy with the exact result for much longer, diverging only at around 70 or 60 fs, respectively. To explore this curiosity, we can compare all elements of the density matrix rather than just a single population; this can be achieved with the complex analogue of the dot product to quantify the similarity of an approximately propagated vectorised density matrix |**ρ**^LVC^⟩ to one propagated using CASSCF-SO Hamiltonians |**ρ**^ab initio^⟩
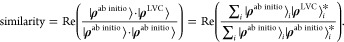
9The decay of the similarity metric from 1 is a measure of how rapidly an approximate trajectory diverges from the ab initio case, considering the phase of off-diagonal elements as well as their magnitude. Deviations in the population dynamics are first observed for **H**_100_^LVC^ and **H**_40_^LVC^ when their similarity to the ideal trajectory falls below 0.998 (Figure 5), suggesting that the apparent better performance of **H**_100_^LVC^ and **H**_200_^LVC^ is simply an artifact of the random nature of the truncation error, which leads to some populations being more affected than others.

The performance of the LVC method will vary when different terms dominate the molecular Hamiltonian, as well as when the rate at which geometry changes during an MD trajectory varies. A similar methodology should thus be applied to benchmark the method for any given system, and we suggest that maintaining errors <1% is a good starting point; significant deviations in the spin dynamics appear when the errors reach ∼2%.

While the Hamiltonians generated by the LVC method have been shown to be usable for spin dynamics simulations, its viability as a cost saving measure depends on its computational expense relative to an approach based on many electronic structure calculations. Unfortunately, in the present context, the LVC parametrization itself is expensive enough that quantitatively accurate electron spin dynamics can be simulated more cheaply using a CASSCF-SO calculation at each MD time step. That is, one LVC parametrization is more expensive than 10 explicit CASSCF-SO calculations in this particular example. There are two components that hence limit this approach: the first is the calculation of the gradients and NACs at the CASSCF level, which makes the computational effort greater. However, this may not be the case for every system–the number of gradients and NACs to be computed scales quadratically at leading order with the number of relevant spin-free states. In this case Dy^3+^ requires the consideration of 18 states for a reasonable description of its electronic structure, where other compounds can require far fewer. For example, the same molecular structure but examining Ce^3+^ or Yb^3+^ would require only seven states. To quantify this difference, we performed an LVC parametrization for [YbL^1^] and found it required only 13% of the core hours needed for the average [DyL^1^] parametrization, where a CASSCF-SO calculation for the Yb^3+^ compound, using an active space of 13 electrons in the seven 4f orbitals, was 64% as expensive as the average equivalent calculation for Dy^3+^. The LVC approach is still too expensive to be used for the quantitative spin dynamics of [YbL^1^], but for the qualitatively accurate dynamics achieved with **H**_40_^LVC^ it would break even.

The second component that makes the present example difficult is the need to parametrize a new LVC model every 10 fs: perhaps other molecules may not be so sensitive to changes in structure. Indeed, [DyL^1^] has been shown to feature an electronic structure that is exquisitely sensitive to molecular geometry^7,11,12^ – as such, it is possible that similarly high truncation errors are reached more slowly for generic lanthanide complexes. The values of gradients and NACs varied from −0.133 to 0.196 *E*_h_*a*_0_^–1^ during the 100 fs investigated here, while those for an optimized^53^ structure of [DyDOTA]^−^ have a range of only −0.0364 to 0.0356 *E*_h_*a*_0_^–1^ (see SI for details). However, a similarly optimized structure of [DyL^1^] had a range of −0.0421 to 0.0472 *E*_h_*a*_0_^–1^, suggesting that this effect may be exaggerated by the optimized structure being close to a minimum on the potential energy surfaces of at least some states. While the behavior of higher order derivatives is not known, they are likely to also be larger for [DyL^1^]. For a system without such unusual geometry dependence and with a more computationally facile metal, such as the Tb^3+^ PARASHIFT agent proposed by Finney and co-workers^13^ (Scheme 1 of that study), it may well be possible to perform comparatively inexpensive electron spin dynamics using an LVC-based approach.

**Figure 6:**
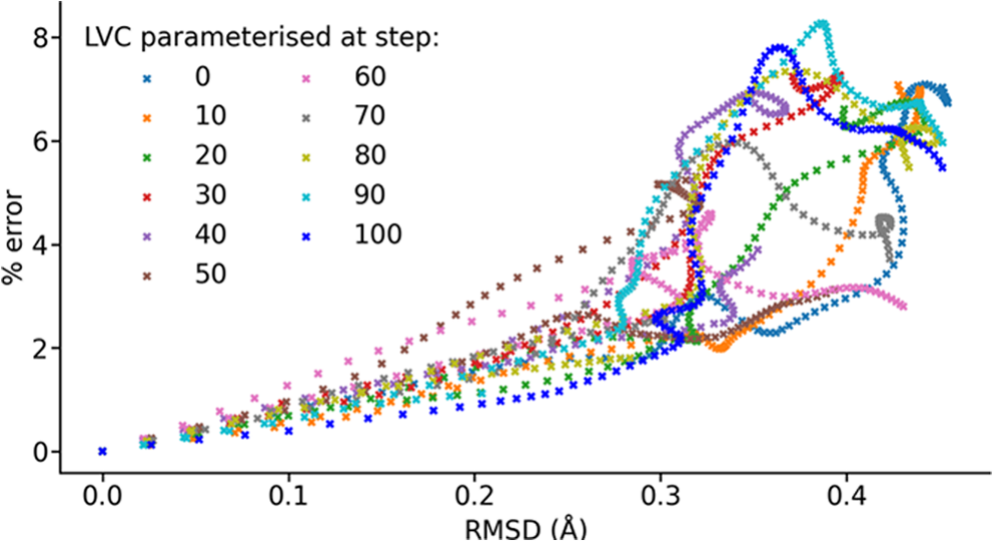
Root-mean-square deviation between the geometry an LVC model was parametrized at and that at which it is used to generate a spin Hamiltonian (found after alignment using the Kabsch algorithm) against the truncation error present in that spin Hamiltonian.

Other problems may also exhibit changes in geometry that occur less rapidly relative to the time scale of spin dynamics, in which case LVC parametrizations would be expected to remain more accurate for longer. The use of LVC parametrizations at regular intervals is questionable in these cases, where errors may be low but not reliably similar at relatively large geometric deviations–some kind of adjustable interval size may be necessary to achieve the smallest number of parametrizations for a given maximum error. The obvious metric to use for this would be root-mean-square deviation (RMSD) of atomic positions, calculated following Kabsch rotation,^54^ which indeed appears to be linearly correlated with the truncation error until RMSD exceeds 0.15 Å (Figure 6). A more approximate linear relation may be applicable until RMSD is over 0.25 Å. We investigated a variable interval approach for our 100 fs window, whereby the current parametrization would be discarded when RMSD relative to the parametrization geometry exceeds a set threshold. A new parametrization was then performed at the new geometry, and subsequent RMSD calculations performed against the new reference. Reparameterisation is necessary only at every other incident of the RMSD threshold being exceeded because the LVC model can approximate Hamiltonians backward through an MD trajectory as effectively as it can forward. If the threshold is set to 0.1 Å, approximately corresponding to the 1% error reached under **H**_10_^LVC^, the timesteps chosen for parametrization are identical other than 61 fs being used instead of 60 fs. If a threshold of 0.25 Å is used, however, parametrizations are required at 0, 28, 59, and 88 fs, demonstrating that this approach has a more significant impact if larger geometric deviations are acceptable. It is likely to be effective within the regime of linear correlation between truncation error and RMSD, as long as the resulting distribution of maximum errors due to different parametrizations can be tolerated. In fact, a similar distribution of maximum errors is also present when using a fixed interval.

Finally, although requiring computation of second derivatives, a quadratic vibronic coupling model has the potential to expand the range of geometries accurate Hamiltonians can be computed for to cover all or most of an MD trajectory. This would have to use expensive numerical differentiation schemes, but could be made feasible by considering a subset of second derivatives, for example only those involving the motion of two donor atoms, or of two atoms with first derivatives above a certain threshold.

## 4 Conclusions

The LVC model has been demonstrated to be a viable source of approximate Hamiltonians along an MD trajectory. We have shown that these are accurate enough in the present case study to achieve quantitative agreement for electron spin dynamics simulations when LVC models are parametrized every 10 fs. Alternatively, if only qualitative accuracy is needed, computational effort can be significantly reduced (e.g., perhaps by a factor of 2 to four, depending on the application domain) by increasing the interval between parametrizations. A benchmarking process has been developed, and should be considered when applying the model to new systems. The computational expense of parametrizing the LVC model is a major consideration when evaluating its usefulness in a given case; it is most appropriate for those with relatively few relevant spin-free states, slow changes in geometry and potentially with limited dependence of electronic structure on geometry. The LVC model is a promising tool for approximating molecular Hamiltonians at different molecular geometries, and should be considered wherever such information is required, for instance when performing chemically detailed electron spin dynamics simulations.

## Supporting Information Available

Methodology for lanthanide complex geometry optimizations; errors in individual spin Hamiltonian terms as a function of time; spin Hamiltonian parameters as functions of time; density matrix trace as a function of time (PDF)
