# Correction to “Approximate Hamiltonians from a Linear Vibronic Coupling Model for Solution-Phase Spin Dynamics”

**DOI:** 10.1021/acs.jctc.5c00683

**Published:** 2025-05-15

**Authors:** Toby R. C. Thompson, Jakob K. Staab, Nicholas F. Chilton

In our recent publication, we examined how a LVC model may be used across an MD trajectory to approximate CASSCF-quality Hamiltonians at lower cost.^1^ A key part is that the molecular geometry of each subsequent frame in the MD trajectory is rotated to match the geometry where the LVC model is defined to minimize the Euclidean distance and hence minimize the truncation error of the linear model. The implementation of the Kabsch algorithm^2^ used to align molecular geometries was incorrect, giving an incorrect molecular rotation, making the truncation error unnecessarily large. After repeating our computations with a correct implementation of the algorithm, our conclusions remain fundamentally the same. However, several figures require amending. Figures 2, 3, 4, 5, and 6 in this Addition/Correction replace those with the same numbers in the original published paper. The corrected errors are approximately half those seen in the original text. The maximum error appears to be unchanged. Several figures in the Supporting Information have also been updated, but the conclusions drawn from them remain the same.

These lower errors lead to a slightly more positive appraisal of the LVC model as a tool for MD-driven spin dynamics than what is described in our original report. Quantitatively accurate dynamics can in fact be achieved with LVC parametrizations performed every 20 fs. Qualitatively correct behavior is seen for separations of up to 100 fs. Despite this, our finding that the LVC approach is too expensive to be used for [DyL^1^] remains valid. However, for a potential qualitative spin dynamics study of a Yb^3+^ complex, we now find that the LVC model would allow for computational savings of ∼50% versus an approach with CASSCF-SO calculations performed at every time step.

**Figure 2:**
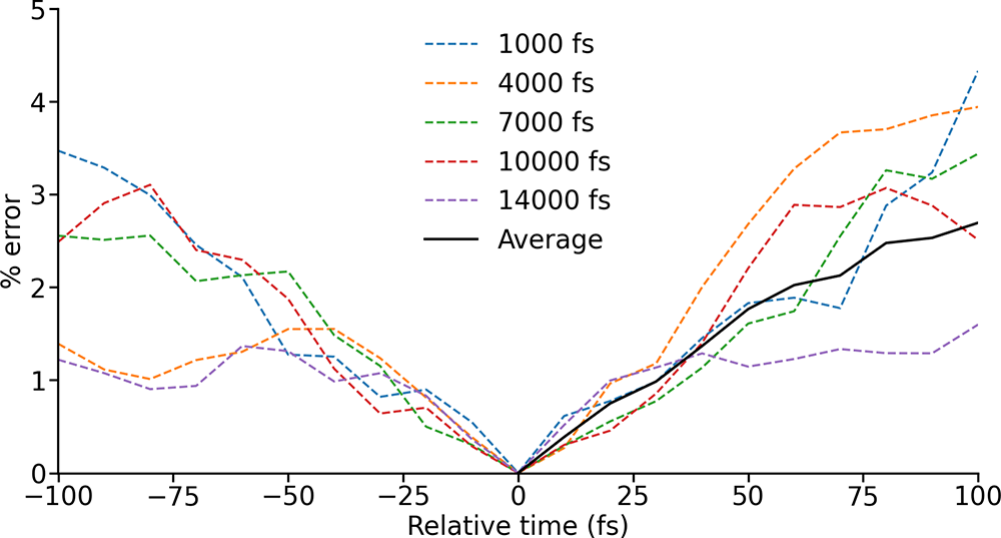
Error in an LVC-generated spin Hamiltonian as a function of the separation between the parametrization (relative time 0 fs) and the time at which the spin Hamiltonian is evaluated. This is plotted with the LVC parametrization carried out at a range of different times throughout an MD trajectory and, as an average, taking into account both positive and negative relative time.

**Figure 3:**
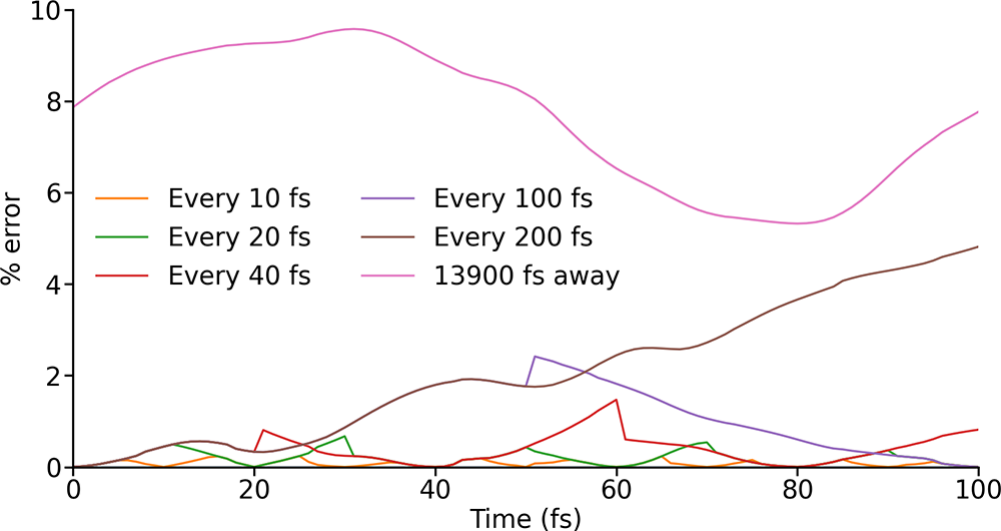
Error in an LVC-generated spin Hamiltonian over 100 fs with LVC parametrizations carried out at different intervals. 0 fs is the beginning of the error trajectory and the point at which the LVC model is first parametrized. In one case a single LVC parametrization is carried out at 13900 fs.

**Figure 4:**
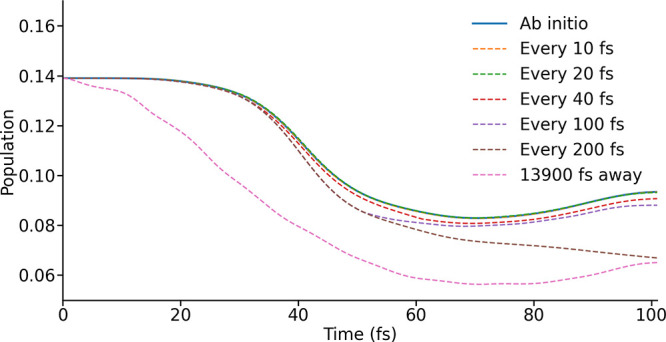
Population trajectories for one of the ground eigenstates of the initial spin Hamiltonian, found using spin Hamiltonians from *ab initio* calculations and the LVC model parametrized at different intervals. In one case a single LVC parametrization, carried out at 13900 fs, is used.

**Figure 5:**
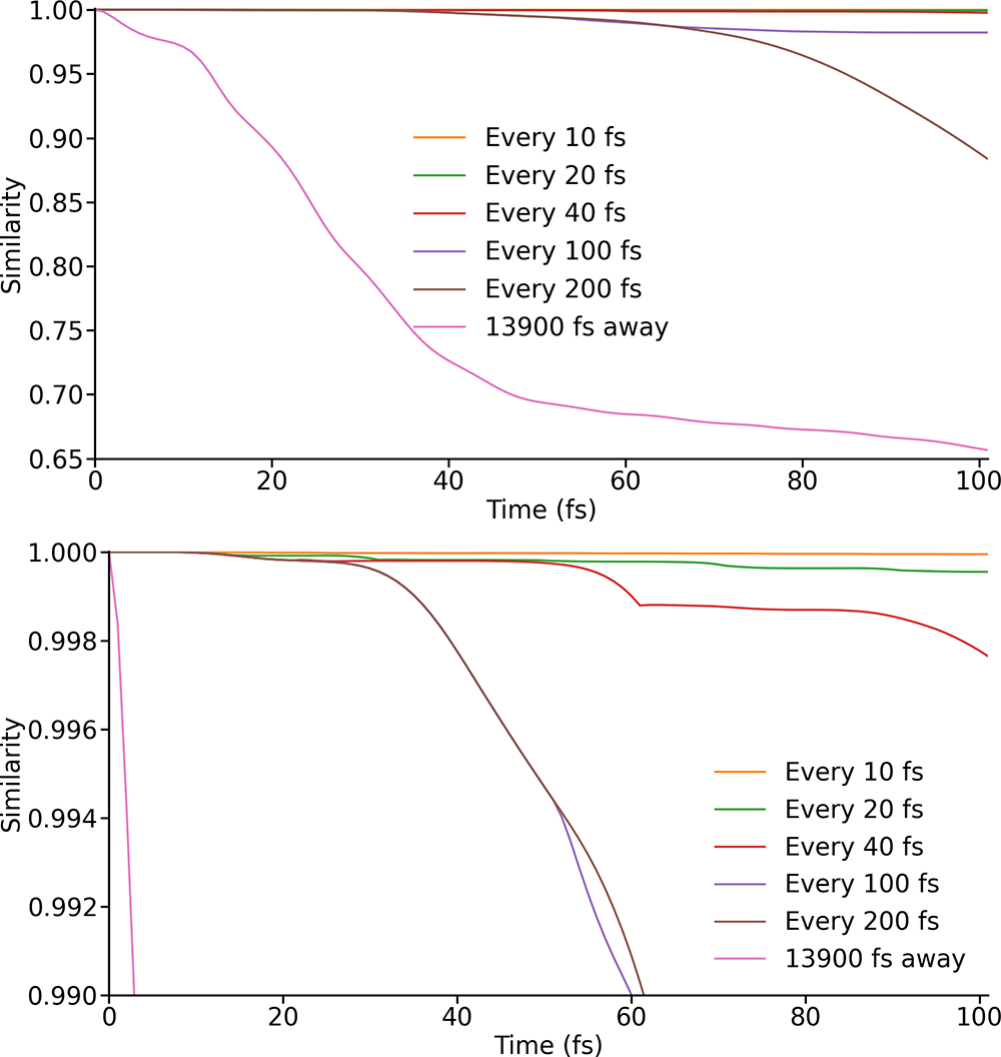
Similarity between the exactly propagated density matrix and those propagated using spin Hamiltonians from LVC models parametrized at different intervals. In one case a single LVC parametrization, carried out at 13900 fs, is used. The lower image has been scaled to demonstrate the divergence of even the most accurate LVC-propagated trajectories.

**Figure 6:**
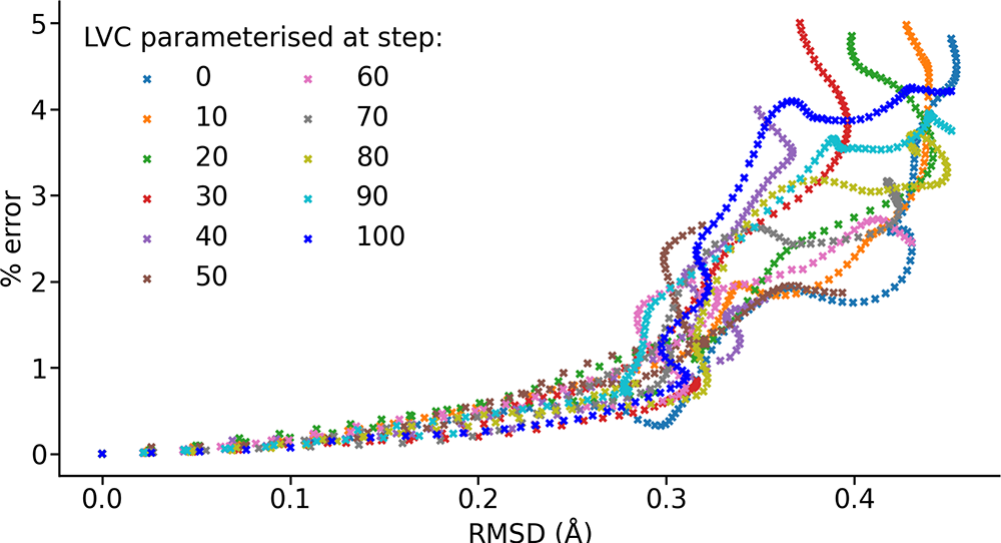
Root-mean-square deviation between the geometry an LVC model was parametrized at and that at which it is used to generate a spin Hamiltonian (found after alignment using the Kabsch algorithm) against the truncation error present in that spin Hamiltonian.

## Supplementary Material



## Notes

Corrected Supporting Information file (PDF)
