# Supplementary material for: Approximate Hamiltonians from a Linear Vibronic Coupling Model for Solution-Phase Spin Dynamics
Source: J Chem Theory Comput. 2025 Jan 17;21(3):1222–9. doi: 10.1021/acs.jctc.4c01437 (PMC11823414; doi:10.1021/acs.jctc.4c01437)
Supplement: Supplementary file 1 — ct4c01437_si_001.pdf [file ct4c01437_si_001.pdf]

Supporting Information: Approximate Hamiltonians from a Linear Vibronic Coupling Model for Solution-Phase Spin Dynamics

Toby R. C. Thompson,[†] Jakob K. Staab,[†,¶] and Nicholas F. Chilton[*,†,‡]

†*Department of Chemistry, The University of Manchester, Manchester M13 9PL, U.K.*

‡*Research School of Chemistry, Australian National University, Canberra, Australian Capital Territory 2601, Australia*

¶*Department of Chemistry "Ugo Schiff", INSTM Research Unit, Università degli Studi di Firenze, 50019 Sesto Fiorentino, Italy*

E-mail: nicholas.chilton@anu.edu.au

Geometry Optimisations Using Density Functional Theory

Geometry optimisations employing restricted Kohn-Sham density functional theory were performed in the Orca 6.0.1 software[S1] using the PBE exchange-correlation functional[S2] with the D3 dispersion correction[S3] and Becke-Johnson damping.[S4] The cc-pVDZ basis set[S5] was employed, along with the RI-J approximation[S6] for Coulomb integrals. An f-in-core pseudopotential of the Stuttgart group,[S7] alongside the corresponding optimised basis set, was applied to the metal.

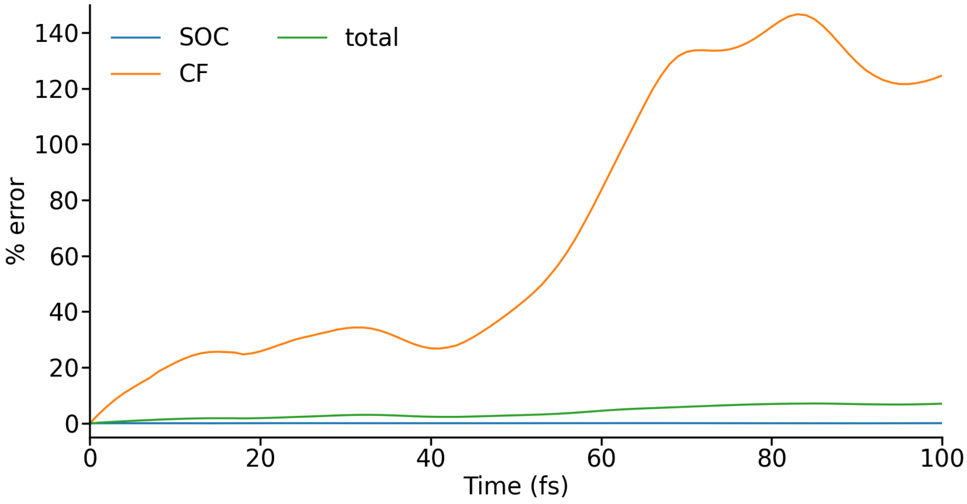

Figure S1: The error in different contributions to an LVC-generated spin Hamiltonian over 100 fs. A single LVC parameterisation was carried out at 0 fs.

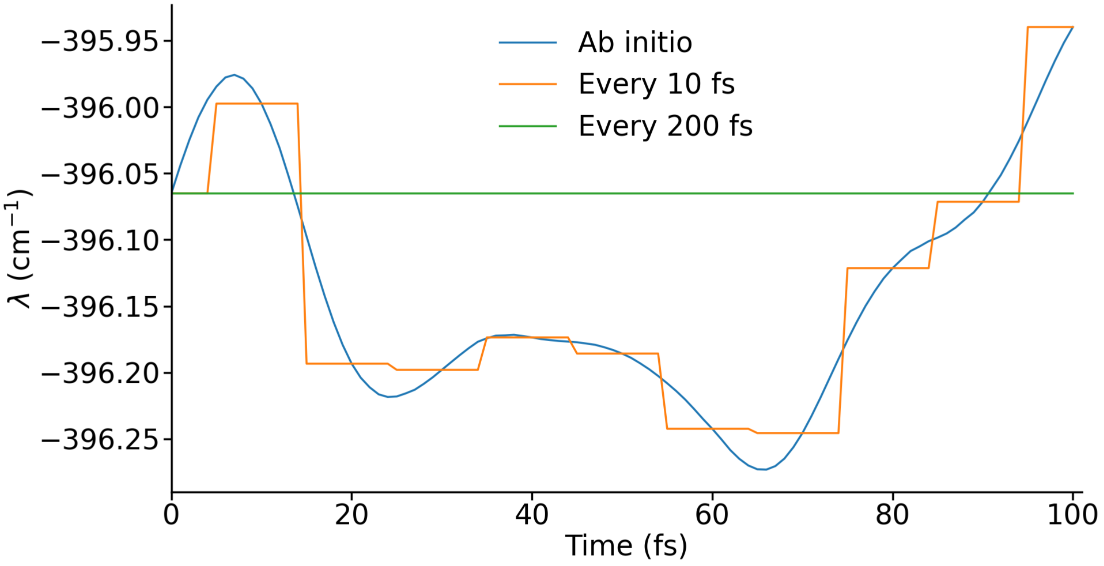

Figure S2: The SOC parameter λ over 100 fs of an AIMD simulation. Projected from *ab initio* calculations and the LVC model with parameterisations at different intervals.

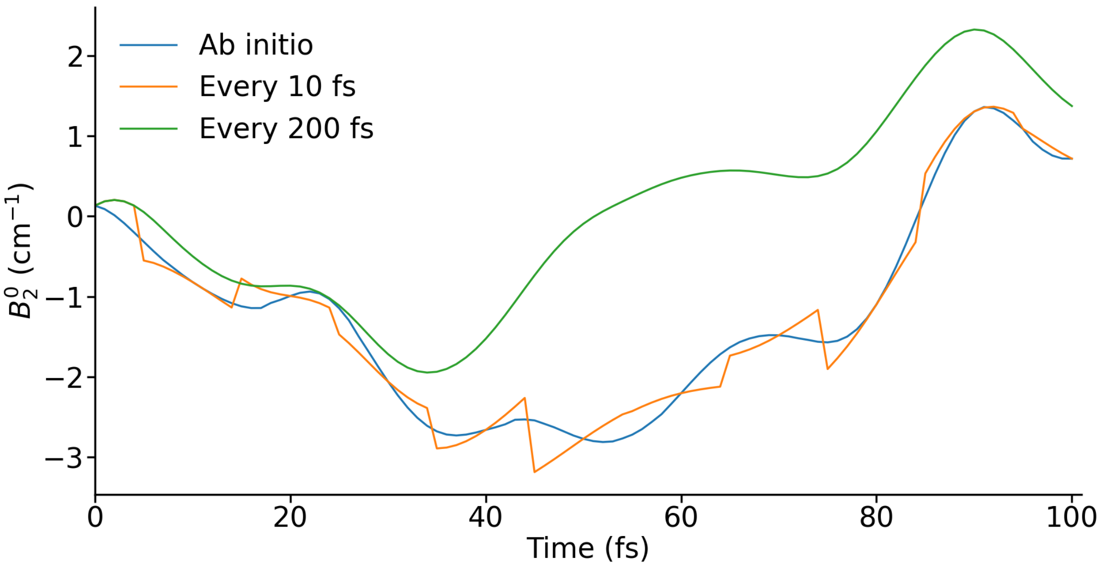

Figure S3: The crystal field parameter B_2^0 over 100 fs of an AIMD simulation. Projected from *ab initio* calculations and the LVC model with parameterisations at different intervals.

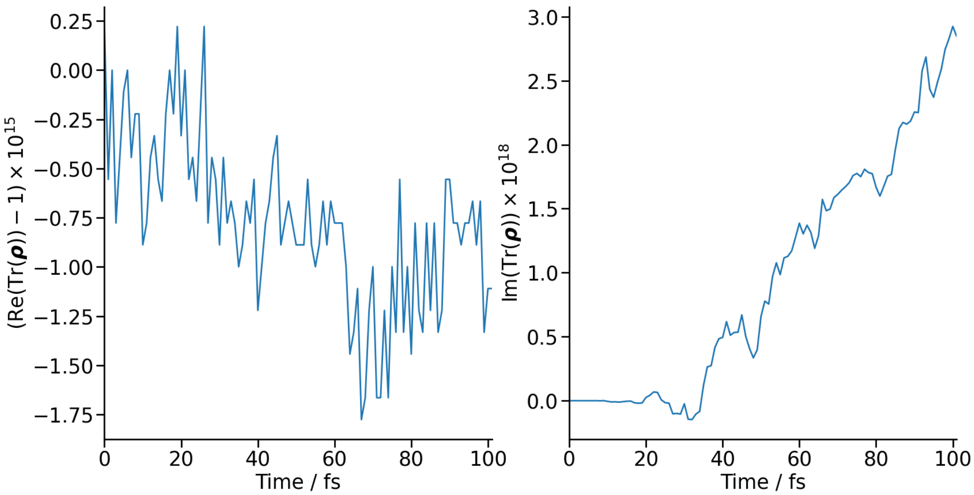

Figure S4: The components of the trace of the density matrix over a 100 fs spin dynamics simulation with Hamiltonians generated by the LVC method with parameterisations every 10 fs. $\mathrm{Tr}(\boldsymbol{\rho}(t)) = 1 + 0i$ must be maintained for any successful spin dynamics simulation.

References

(S1) Neese, F.; Wennmohs, F.; Becker, U.; Riplinger, C. The ORCA quantum chemistry program package. *J. Chem. Phys.* **2020**, *152*, 224108.

(S2) Perdew, J. P.; Burke, K.; Ernzerhof, M. Generalized Gradient Approximation Made Simple. *Phys. Rev. Lett.* **1996**, *77*, 3865–3868.

(S3) Grimme, S.; Antony, J.; Ehrlich, S.; Krieg, H. A consistent and accurate ab initio parametrization of density functional dispersion correction (DFT-D) for the 94 elements H-Pu. *J. Chem. Phys.* **2010**, *132*, 154104.

(S4) Johnson, E. R.; Becke, A. D. A post-Hartree-Fock model of intermolecular interactions: Inclusion of higher-order corrections. *J. Chem. Phys.* **2006**, *124*, 174104.

(S5) Dunning, T. H. Gaussian basis sets for use in correlated molecular calculations. I. The atoms boron through neon and hydrogen. *J. Chem. Phys.* **1989**, *90*, 1007–1023.

(S6) Neese, F. An improvement of the resolution of the identity approximation for the formation of the Coulomb matrix. *J. Comp. Chem.* **2003**, *24*, 1740–1747.

(S7) Dolg, M.; Stoll, H.; Savin, A.; Preuss, H. Energy-adjusted pseudopotentials for the rare earth elements. *Theor. Chim. Acta* **1989**, *75*, 173–194.
